# Vestibular Interactions in the Thalamus

**DOI:** 10.3389/fncir.2015.00079

**Published:** 2015-12-02

**Authors:** Rajiv Wijesinghe, Dario A. Protti, Aaron J. Camp

**Affiliations:** ^1^Sensory Systems and Integration Laboratory, Sydney Medical School, Discipline of Biomedical Science, University of SydneySydney, NSW, Australia; ^2^Vision Laboratory, Sydney Medical School, Discipline of Physiology, University of SydneySydney, NSW, Australia

**Keywords:** vestibular, thalamus, LGN, multisensory integration, vestibular nuclei

## Abstract

It has long been known that the vast majority of all information en route to the cerebral cortex must first pass through the thalamus. The long held view that the thalamus serves as a simple hi fidelity relay station for sensory information to the cortex, however, has over recent years been dispelled. Indeed, multiple projections from the vestibular nuclei to thalamic nuclei (including the ventrobasal nuclei, and the geniculate bodies)- regions typically associated with other modalities- have been described. Further, some thalamic neurons have been shown to respond to stimuli presented from across sensory modalities. For example, neurons in the rat anterodorsal and laterodorsal nuclei of the thalamus respond to visual, vestibular, proprioceptive and somatosensory stimuli and integrate this information to compute heading within the environment. Together, these findings imply that the thalamus serves crucial integrative functions, at least in regard to vestibular processing, beyond that imparted by a “simple” relay. In this mini review we outline the vestibular inputs to the thalamus and provide some clinical context for vestibular interactions in the thalamus. We then focus on how vestibular inputs interact with other sensory systems and discuss the multisensory integration properties of the thalamus.

## Introduction

The vestibular system differs from the other primary sensory systems in a number of fundamental ways. Most sensory systems are organized in a linear fashion, where peripheral organ fibers project primarily through a modality-specific thalamic nucleus (for example LGN for the visual system, MGN for the auditory system) and only then onto their respective cortical or subcortical targets. These ordered projections through the thalamus create a sensory map that closely matches that created in the periphery, and this tends to be maintained by downstream thalamocortical projections [for review, see Jones (1985)]. This organization, along with ordered corticothalamic feedback mechanisms, allows the thalamus to play filtering and modulatory roles in sensory processing (see Sherman, 2007 and references within). Hair cells within the vestibular epithelia are oriented such that they are direction-selective (Lindeman, 1969), however when afferent fibers that relay information from these cells in the semicircular canals, utricle and saccule all converge onto the brainstem vestibular nuclei this signal then becomes more complex. For example, some thalamic neurons receiving vestibular information appear to selectively fire when the organism’s head is orientated in a specific direction within the environment, much like a compass (for comprehensive review, see Wiener and Taube, 2005). The brainstem vestibular nuclei project directly to the thalamus, however there is no dedicated vestibular thalamic nucleus that contains a direction or orientation selective map. Further, vestibular thalamic projections are widely distributed to subcortical (Lai et al., 2000), cerebellar (Kotchabhakdi and Walberg, 1978) and cortical (de Waele et al., 2001; Miyamoto et al., 2005) regions that have been shown to be involved in vestibular processing.

Cortical processing of vestibular information is poorly understood compared to other sense modalities such as vision, audition and touch. The perceptual correlate of vestibular activation does not usually correspond to a unimodal sensation, as it occurs with vision and audition. Natural activation of the vestibular system due to head motion and locomotion typically involves activation of visual, vestibular and somatosensory systems and consequently the resulting perceptual correlate are sensations that involve visual, vestibular and somatosensory characteristics. Vestibular-responsive neurons were initially described in somatosensory cortex in areas 3a (Grusser et al., 1990) and area 2v (Buttner and Buettner, 1978). More recently, head direction neurons responsive to vestibular stimulation have also been identified in regions within the classical Papez circuit. Whether signals from different modalities reach these regions independently or they converge at earlier stages is not completely understood yet. There is, however, evidence consistent with integration at the level of the thalamus.

These fundamental observations highlight that the vestibular system has a unique structure amongst the sensory systems, and raises some important questions. In particular, what role does the thalamus play in vestibular processing? In this mini-review we will explore the current understanding of vestibular thalamic function. In the first section, we summarize anatomical studies of vestibulothalamic projections, and suggest that they may be organized into modality-specific processing streams that permit multisensory integration. In the second section, we analyze physiological studies exploring how vestibular thalamic neurons respond to vestibular information, and clinical observations that give us insights into vestibular processing within the thalamus. In the final section, we make hypotheses about the mechanisms of multisensory integration using the vestibular system as an example, and suggest that the thalamus is well placed to represent a unique subcortical locus of multisensory integration.

## Vestibulothalamic Projections

Peripheral vestibular stimulation has been shown to cause strong activation within the thalamus (Deecke et al., 1974; Buttner and Henn, 1976; Blum et al., 1979). Tracing studies performed in rat (Shiroyama et al., 1999), cat (Kotchabhakdi et al., 1980), and monkey (Meng et al., 2007), as well as radiological investigations in humans (Kirsch et al., 2015) have demonstrated multiple projections from vestibular nuclei to the thalamus. The primary thalamic targets are the ventrobasal, ventrolateral and intralaminar nuclei and the geniculate bodies (for a recent review, see Lopez and Blanke, 2011). Many of these nuclei are not specific to the vestibular system, and therefore receive inputs from a number of different peripheral sensory and cortical regions. There is some evidence to suggest that these vestibulothalamic circuits may form discrete, functionally specialized pathways that integrate vestibular with other modality-specific signals within the thalamus (Lopez and Blanke, 2011).

*Inter alia*, the ventrobasal nuclei (ventral posterolateral, VPL; ventral posteromedial, VPM; and ventral intermediate, VI) receive dominant vestibular inputs from bilateral superior vestibular nucleus (SuVN), and contralateral medial vestibular nucleus (MVN) via the medial longitudinal fasciculus (MLF; Nagata, 1986; Matesz et al., 2002). VPM neurons have been shown to respond directly to vestibular nerve stimulation, as well as simulated translational and rotational movements (Marlinski and Mccrea, 2008). These nuclei in turn project to well known vestibular cortical areas such as the anterior suprasylvian cortex (Deecke et al., 1979), posterior cruciate region and the intraparietal sulcus (for review, see Brandt and Dieterich, 1999). The ventral posterior nuclei (VP or VPP) in particular is the source of the major projection to a well studied vestibular cortical region in the macaque monkey, the PIVC (Akbarian et al., 1992). However, it has been shown that some posterior thalamic nuclei such as the VP are also activated during deep somatic and cutaneous stimulation (Deecke et al., 1977). In addition, the VPI projects directly to primary somatosensory cortex (Deecke et al., 1974), as well as vestibular regions of the secondary association somatosensory cortex (3aV) and posterior parietal cortex (Matsuzaki et al., 2004). These observations show that the thalamus contains the neural circuits necessary to mediate the integration of vestibular and somatosensory signals.

**Figure 1:**
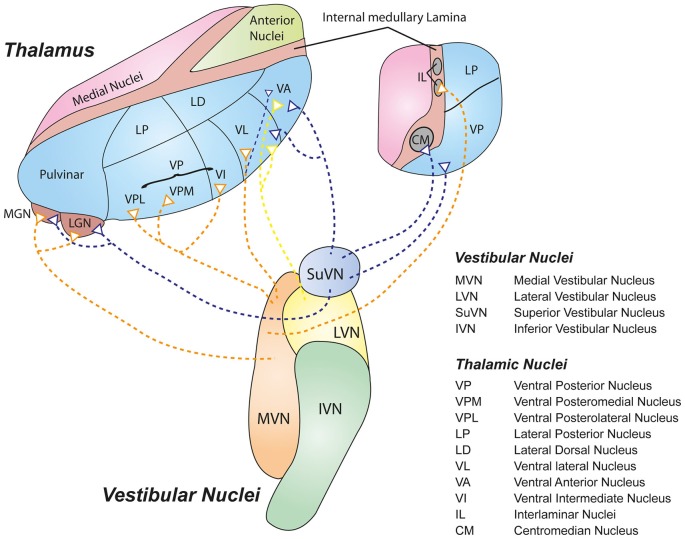
**Schematic representation of some of the major vestibulothalamic projections.** Defined projections from the four main vestibular nuclei to their thalamic targets are shown. Note that the vast majority of vestibulothalamic projections originate in the medial vestibular nucleus (MVN; orange) and SuVN (mauve), and terminate in the lateral nuclei of the thalamus (Blue).

The ventrolateral nuclei (ventral anterior, VA; ventral lateral, VL) receive inputs primarily from LVN, MVN and SuVN via the medial longitudinal fasciculus (MLF; Maciewicz et al., 1982; Nakano et al., 1985; Nagata, 1986). Interestingly, the VL also receives inputs from vestibular cerebellar nuclei such as the fastigial and dentate nuclei via the superior cerebellar peduncle (Sawyer et al., 1994a,b), while the VA receives strong inputs from the basal ganglia (Percheron et al., 1996). The VA-VL complex projects to primary motor and premotor cortices (Brodmann 4 and 6), suggesting that this circuit may represent a major vestibulomotor pathway. The intralaminar nuclei (CL or PFN in rat, CM) receive inputs primarily from ipsilateral MVN, SuVN and spinal vestibular nucleus (SpVN; Magnin and Kennedy, 1979; Matsuo et al., 1994). Specifically, the PFN has been shown to be a crucial synapse for a vestibulo-thalamo-striatal pathway proposed to be involved in controlling body and limb movements (for review, see Stiles and Smith, 2015). Further, the geniculate bodies (MGN, LGN, SGN) receive inputs from DVN, SuVN and MVN (Kotchabhakdi et al., 1980; Mergner et al., 1981). Despite their primary role in auditory and visual signalling (MGN and LGN respectively), these centers have also been shown to respond to vestibular signals (Liedgren and Schwarz, 1976; Roucoux-Hanus and Boisacq-Schepens, 1977), suggesting that these thalamic nuclei may participate in subcortical multi-sensory integration. Figure 1 illustrates some of the major pathways connecting the central vestibular nuclei and the thalamus. Note that most of the inputs terminate in the lateral nuclei of the thalamus.

## Thalamic Vestibular Processing

The vestibular nuclei project heavily to the spinal cord and cerebellum, and these pathways mediate important reflexes that maintain postural control and motor learning (Granit and Pompeiano, 1979; Pompeiano and Allum, 1988). As is evident from the discussion above, there are multiple, divergent vestibular projections that course through the thalamus. However there is limited information about what role the thalamus plays (in concert with the vestibular system) in postural control, and only recently has work begun to delineate its fundamental vestibular processing characteristics.

Clinical observations provide some useful insights into the thalamic contribution to vestibular processing. Postural disorders are increasingly well recognized in patients recovering from strokes, some of which cannot solely be attributed to the unilateral loss of muscular control. For example, Pusher syndrome is an active righting movement observed following strokes where the patient “push[es] actively with non-paretic extremities to the side contralateral to the brain lesion” (Karnath et al., 2005). The lesion induces an abnormality in the perception of the body’s alignment in relation to gravity, and has been described in patients with circumscribed posterior thalamic lesions as well as other adjacent brain structures (Karnath et al., 2005). Specifically, patients with pusher syndrome perceive their body to be positioned vertically despite approximately 18 degrees of tilt of the subjective postural vertical in the roll plane (for review see Karnath, 2007). Interestingly the subjective visual vertical appears remarkably undisturbed in pusher syndrome (Karnath et al., 2000). The observation that cortical infarcts are also capable of inducing this syndrome (Johannsen et al., 2006; Karnath et al., 2008) or other perturbations of subjective visual vertical (Baier et al., 2012) suggests that the posterior thalamus is important but not unique in generating this perception of verticality. Another interesting insight comes from studies of patients suffering from vestibular migraine, a central vestibular disorder characterized by an abnormal vestibular percept without peripheral vestibulopathy (for recent review, see Stolte et al., 2015). Functional magnetic resonance imaging (fMRI) studies in these patients during vestibular stimulation demonstrated increased BOLD signal within the thalamus compared to both healthy controls and migraineurs with aura (Russo et al., 2014). These observations demonstrate that the thalamus plays in integral role in the generation of complex vestibular percepts, and that abnormalities in thalamic processing may account for the distortion of these percepts observed in certain clinical situations.

**Figure 2:**
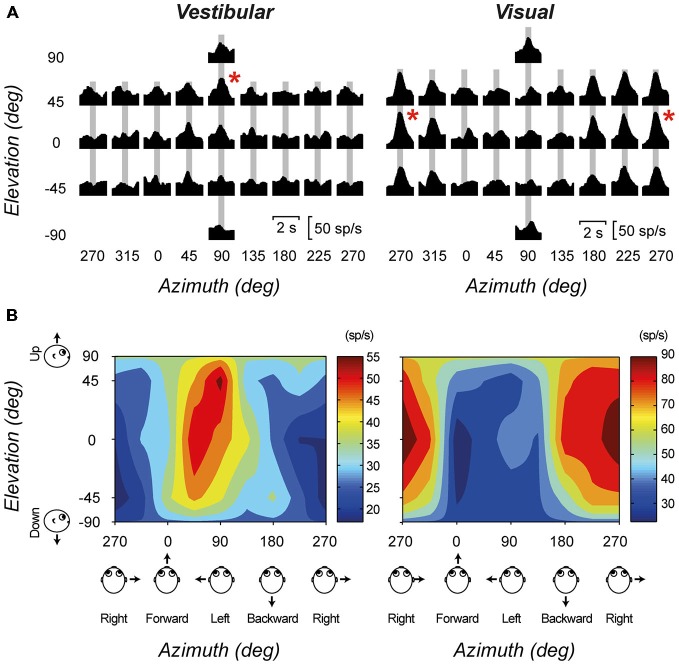
**An example cell with significant tuning during both the visual (optic flow) and vestibular stimulus conditions. (A)** Response PSTHs for each of 26 directions during translation. Red asterisks mark the maximum response directions. The corresponding peak-times are marked by gray bars. **(B)** The corresponding color contour tuning (at peak time) for each stimulus condition. Figure modified from Meng and Angelaki (2010) with permission from the Journal of Neurophysiology.

A number of *in vivo* electrophysiology studies have demonstrated the vestibular evoked responses of thalamic neurons. Meng et al. (2007) recorded the responses of thalamic neurons in the ventral posterior and ventral lateral thalamic nuclei of macaque monkeys. In general, they found that vestibular-responsive thalamic neurons show mixed selectivity for rotations and translations within different planes, however they tend to be more responsive to translations within the yaw plane. Subsequently, a small subset of thalamic neurons was found to be responsive to both vestibular and visual inputs (Meng and Angelaki, 2010). Figure 2 shows the translation direction tuning to both visual and vestibular stimulation for one example multisensory neuron. Similar responses were also observed for rotational stimuli. In addition, experiments by Marlinski and colleagues demonstrated that neurons tended to respond either to ispi- or contralateral rotation, with no neurons displaying bidirectional sensitivity (Marlinski and Mccrea, 2008). Experiments performed in both light and dark environments demonstrate that the vestibular signal in the thalamus is independent of visual signals, however the addition of visual information does increase the sensitivity of these neurons to incoming inputs. Both groups showed a large range of responsiveness to rotational stimulation, in both amplitude and phase. Their experiments demonstrated a large number of vestibular responsive areas within the thalamus, outside of the classically described vestibulothalamic projection zones. Interestingly, Marlinski and colleagues demonstrated small clusters of vestibular-responsive neurons within the VP nucleus, and postulate that these may be analog to the discrete regions seen within the macaque thalamus that project to specific vestibular-specific cortical regions (Marlinski and Mccrea, 2008).

Few investigators have studied the intrinsic properties of neurons within the thalamus that receive vestibular inputs. One recent study by Kulkarni and colleagues examined the properties of rat anterodorsal thalamic neurons in the presence of synaptic blockade (Kulkarni et al., 2011). They found that single neurons were able to sustain long periods of regular firing over many minutes with minimal adaptation. This response was particularly evident when induced by hyperpolarizing as opposed to depolarizing pulses. Work studying the synaptic profile of anterodorsal thalamic neurons suggests that they are characteristic of those within a first order relay nucleus, with a driver input from the mammillary bodies and modulatory input from cortical feedback (Petrof and Sherman, 2009). It would be interesting to see whether these neurons also display similar output profiles to other first-order relay thalamocortical neurons such as those seen in the lateral geniculate nucleus (Gutierrez et al., 2001; Wijesinghe et al., 2013).

## Multisensory Integration in the Thalamus

Perceptually, the vestibular system is integral in generating higher order sensory phenomena that require input from other sensory systems. For example, creating the sense of the visual vertical requires input from both visual and vestibular neurons (Vingerhoets et al., 2009); postural stability requires input from proprioceptive neurons and vestibular neurons (Bronstein, 1999). Studies have also unexpectedly suggested a role for the vestibular system in cognitive domains such as memory and learning (Smith and Zheng, 2013) and body representation (Mast et al., 2014). As outlined above, thalamic nuclei receiving vestibular afferents are also heavily involved in the processing of information from other sensory systems. This raises the possibility that the thalamus may serve as an important locus for multisensory integration. Indeed, a recent study shows tantalizing evidence that driver (primary and secondary vestibular) and modulatory (visual) inputs onto cerebellar granule cells have different strength and temporal course. In addition, when these inputs are co-activated they produce an enhanced response with a characteristic first spike latency that allows temporal coding of multisensory inputs (Chabrol et al., 2015). It is tempting to speculate, as the authors do, that similar mechanisms may also be involved in the integration of synaptic inputs of different sensory modalities in other thalamic neurons and HD cells that receive vestibular information.

Studies comparing stroke patients with a degree of somatosensory loss to normal controls have suggested that interactions between vestibular and somatosensory information is dependent on the function of the posterolateral thalamus (Barra et al., 2010). Further, psychophysical studies have demonstrated that multisensory integration is impaired in patients with Parkinson’s disease, possibly be due to thalamic dysfunction induced by lack of facilitation by ascending cholinergic systems (Muller et al., 2013). Interestingly, one study reporting on two patients with Parkinson’s following deep brain stimulation of the subthalamic nucleus showed changes in the subjective visual vertical (Mike et al., 2009)- a feature typically associated with peripheral and/or central vestibular dysfunction- again suggestive of vestibular thalamic interactions.

Experimentally, a model system for multisensory integration is the head-direction (HD) system, which contains neurons that encode head orientation in the horizontal (yaw) plane independently of location within an environment (for review, see Taube, 2007). HD neurons have been found in a number of regions within the classical Papez circuit, such as the anterodorsal thalamus, lateral mammillary nuclei, and the retrosplenial and entorhinal cortices (see Wiener and Taube, 2005 and references within for details). The vestibular system plays an integral role in the generation of the HD signal, as demonstrated by behavioral studies assessing spatial navigation while restricting sensory cues or with specific brain regions deactivated (Yoder and Taube, 2014). A recent *in vivo* study analyzing the head-direction cell response to both active and passive movements has shown that vestibular input is necessary and sufficient to generate the HD signal (Shinder and Taube, 2011), highlighting the central role of the vestibular system in this localization pathway.

Thalamic HD neurons have been the focus of recent investigations aiming to elucidate the specific mechanisms behind the HD signal. Using rats on a moving treadmill, Enkhjargal et al. (2014) studied the effect of more complex movements involving multiple frames of reference with conflicting sensory inputs. They found that thalamic HD neurons displayed complex spatial firing patterns that depended on the combinations of facing direction and movement direction; that is, their activity depends on perceived directional heading, optic flow, vestibular and proprioceptive information (Enkhjargal et al., 2014). Interestingly, the directionality seen with the head-direction signal may be dependent on the intrinsic properties of anterodorsal thalamic neurons, specifically their propensity for irregular firing (Tsanov et al., 2014). The finding that the HD signal is also present when motionless (Shinder and Taube, 2014) may suggest that intrinsic mechanisms are capable of maintaining the HD signal once it has been generated. Recent investigations analyzing ensembles of HD-cells within the anterodorsal nucleus and post-subiculum found coherent activations during awake and sleep network states, suggesting that the HD representation may be generated internally within thalamic circuits and modulated by sensory inputs (Peyrache et al., 2015). Another exciting new discovery has shown that parahippocampal grid cell activity may be dependent upon the thalamic head direction signal (Winter et al., 2015), highlighting a central role of the thalamus as a major locus for multisensory integration in the generation of complex perceptual phenomena, particularly in regard to the vestibular contributions to navigation.

## Summary

Our understanding of the vestibular interactions within the thalamus remains nascent, however is growing quite quickly. Clinical and experimental observations are piecing together a clearer picture of how vestibular signals are processed and combined with other sensory signals to form complex representations of the environment and our actions within it. Despite this, the underlying cellular mechanisms that mediate these processes remain unclear. What are the characteristics of individual thalamic neurons in these multisensory pathways? What are the intrinsic properties of these neurons that allow this filtering and integration to take place? What synaptic and intrinsic properties mediate the processing of disparate and conflicting signals from different sensory systems? What is clear, however, is that the thalamus is a point of convergence for signals from essentially all sensory modalities, and that groups of, and even individual neurons within the Thalamus, have the capacity to compute a variety of output functions depending on the signals received. This feature alone is sufficient to clearly define the Thalamus as “beyond a simple relay”.

## Funding

The authors would also like to acknowledge The Support of the Garnett Passe and Rodney Williams Memorial Foundation.

## Conflict of Interest Statement

The authors declare that the research was conducted in the absence of any commercial or financial relationships that could be construed as a potential conflict of interest.

## Abbreviations

CL: Centrolateral Nucleus
CM: Centromedian Nucleus
IL: Interlaminar Nuclei
IVN: Inferior Vestibular Nucleus
LGN: Lateral Geniculate Nucleus
LVN: Lateral Vestibular Nucleus
LP: Lateral Posterior Nucleus
LD: Lateral Dorsal Nucleus
MGN: Medial Geniculate Nucleus
MVN: Medial Vestibular Nucleus
PNF: Parafascicular Nucleus
SpVN: spinal (descending) vestibular Nucleus
SuVN: Superior Vestibular Nucleus
VA: Ventroanterior Nucleus
VI: Ventral Intermediate Nucleus
VL: Ventrolateral Nucleus
VP: Ventral Posterior Nucleus
VPL: Ventral Posterior Lateral Nucleus
VPM: Ventral Posterior Medial Nucleus.
